# It's not just about the park, it's about integration too: why people choose to use or not use urban greenspaces

**DOI:** 10.1186/1479-5868-7-78

**Published:** 2010-10-28

**Authors:** Peter J Seaman, Russell Jones, Anne Ellaway

**Affiliations:** 1 Glasgow Centre for Population Health, 94 Elmbank Street, Glasgow, G2 4DL, UK; 2 Medical Research Council Social and Public Health Sciences Unit, 4 Lilybank Gardens, Glasgow, G12 8RZ, UK

## Abstract

**Background:**

Greenspace has the potential to be a vital resource for promoting healthy living for people in urban areas, offering both opportunities for physical activity and wellbeing. Much research has explored the objectively measurable factors within areas to the end of explaining the role of greenspace access in continuing health inequalities. This paper explores the subjective reasons why people in urban areas choose to use, or not use, local public greenspace.

**Methods:**

In-depth interviews with 24 people living in two areas of Glasgow, United Kingdom were conducted, supplemented with participant photography and participatory methods. Data was thematically categorised to explore subjectively experienced facilitators and barriers to greenspace use in urban areas.

**Results:**

From the perspective of current and potential urban greenspace users, access is revealed to be about more than the physical characteristics of neighbourhoods, greenspace resources or objectively measurable features of walkability and connectivity. Subjectively, the idea of walkability includes perceptions of social cohesion at a community level and the level of felt integration and inclusion by individuals in their communities. Individual's feelings of integration and inclusion potentially mitigate the effects of experiential barriers to urban greenspace access, such as evidence of anti-social behaviour.

**Conclusions:**

We conclude that improving access to greenspace for all in urban communities will require more than providing high quality resources such as parks, footpaths, activities and lighting. Physical availability interacts with community contexts already established and a holistic understanding of access is required. A key cultural component of areas and neighbourhoods is the level of social cohesion, a factor that has the potential to reinforce existing health inequalities through shaping differentiated greenspace access between subgroups of the local population.

## Background

### Understanding greenspaces as health promoting community resources

Although there is now widespread acceptance from studies of neighbourhood effects that where people live affects their health, the underlying mechanisms are only beginning to be understood [1-5]. One mechanism through which local residential areas affect health and wellbeing is through access to health enhancing greenspace. Epidemiological studies have found that the provision of, and access to, greenspaces is an important determinant of health and health inequalities for both individuals and communities and those with greater access to greenspace report better health [6-11]. Mechanisms identified include opportunities to increase cardio-vascular activity, stress reduction and opportunities for interaction for adults and children alike [12-15]. Access to nature and natural environments can also promote mental health by offering psychological and emotional benefits [16,17]. Evidence that green views from hospital beds can assist recovery further highlights how the benefits of greenspace access go beyond opportunities for physical activity [18].

As a consequence of such findings, lack of access to and/or use of urban greenspace become a public health issue for people living in urban areas. With regard to health inequalities between socio-economic groups, greenspace access has been implicated in social inequalities in obesity and overweight, with parks and other urban greenspaces being viewed as important components of community 'opportunity structures' for health [19,20]. Thus conceived, the equalisation of greenspace quality and access between areas is an important public health action to reduce health inequalities [21,22].

The idea of the 'walkability' of communities has become a focus of research seeking to understand inequalities in access and use of available urban greenspace [23-27]. 'Walkable' neighbourhoods are those urban environments in which people feel comfortable, safe, and predisposed to walk. Those defined as "highly walkable" have been described as being characterised by features such high population density, mixed land use (a variety of retail, residential, commercial usage), high connectivity (direct or easy travel routes between destinations as a result of street layout), good pedestrian and cycling facilities (presence of pavements, cycle routes, traffic calming measures) and accessibility (e.g. a variety of destinations or facilities, such as shops, greenspace, transport links) [28,29]. The inventory of factors shaping walkability has developed to exclude the subjective, less easily captured experiences of urban space. This leaves the individual motivations, values and experiences necessary to ensure greenspaces are visited and re-visited relatively unexplored.

Consequently explorations of the relationship between elements of urban infrastructure and health also need to address why people choose, or choose not to use greenspace. While there has been research exploring the connection between physical attributes of local areas and health, most have been conducted using method protocols better placed to capture objective qualities of neighbourhoods such as GIS mapping or surveys. As a result, studies focussing on how places influence health have tended to look at the physical characteristics of neighbourhoods and/or the characteristics of the people who live there [30-32]. Less is known about whether the effects of place may affect individuals differently, in a manner that may further entrench inequalities both within and between areas.

## Methods

The data for this analysis was collected as part of the *Facilitators and Barriers to Greenspace Study *(*FAB Greenspaces*) conducted by a working group of researchers from the Glasgow Centre for Population Health, the Medical Research Council (UK) Social and Public Health Sciences Unit and the local National Health Service (NHS) board (Greater Glasgow and Clyde). The study city, Glasgow, is well-resourced in greenspaces but displays significant inequalities in health both within the city and between other areas of the United Kingdom. The study explored the quality and accessibility of greenspaces across two socially contrasting areas of the city to capture subjective understandings of access and quality. The two localities had been the subject of a longitudinal study and the areas were selected from a continuum of eight socio-residential types in the city of Glasgow, the North West locality being towards the 'better' pole (as measured by census level indicators such as unemployment rates, housing tenure, occupation, and car ownership) and the South West locality towards the 'worse' pole of this continuum, but not at the extremes [33].

The two areas had similar availability of urban greenspace. Indeed the South West area (more deprived) had higher availability (34 percent living within 300 metres of greenspace of two hectares or more compared with 27 percent in the North West). However, when limited to large greenspaces that are managed, the two areas become similar. While there is little change in the North West with the percentage of residents living within 300 metres of managed greenspace greater than 2 hectares decreasing to 25 percent, the shift is more dramatic in the South West, with a drop to 24 percent [34].

### Qualitative methods

For assessing the influence of subjective factors, discussion groups using participatory appraisal techniques and in-depth interviews were utilised. These enabled an in-depth exploration of features of decision-making that stemmed from not only living in a particular area but also how the biographical and social context of individuals led to decisions to use, or not use, available greenspace resources.

#### Participatory Groups

Pilot work with community groups was undertaken to help define the parameters and scope of the data collection in a manner that allowed local people an input. For this we used Participatory Appraisal (PA), a process that allows people to locally determine agendas in consultation. PA is an approach designed to give participants a voice rather than being defined by a rigid set of scientific methods [35]. PA enables people to share their ideas and knowledge about local conditions and allow this expertise to define the nature of local problems. The tools we used were community mapping and H diagrams. Six participatory groups were conducted. Groups were identified and contacted through community hubs. We monitored recruitment to capture a breadth of potential greenspace users, socio-economic statuses, gender and age. The six groups encompassed a pensioners group, a tenants' association and an environmental group (North West), an asylum seeker mothers group, an asylum seeker fathers group and a youth group (South West). The size of groups ranged from 8 to 20. No incentives were offered for participation in the focus groups.

#### Community Mapping

Participants were provided with A1 sheets of paper and coloured markers and asked to visually map their area marking the location of urban greenspace and other community facilities they used. The subjective nature of the maps offered an important counterpoint to more objective data such as GIS as they introduced perceptual barriers such as presence (or evidence of presence) of intimidating others and individual routine based barriers and facilitators of usage.

#### H Diagrams

The groups went into more depth through the use of H diagrams that allow people to list both the positive and negative aspects of their communities related to the use of greenspace and community facilities. We asked people to rate their local community greenspace and community leisure resources on a scale of 1 to 10 (1 equalled poor quality, 10 excellent). This was not intended to be an objective rating but to help focus attention on the issue and to stimulate discussion within the group. After listing the positive and negative features, participants were asked to think about changes that were possible to improve the experience and increase accessibility and usage from their perspectives.

#### Individual in-depth interviews and participant photography

The primary data were collected through 24 interviews in the two localities (12 in each). Interviewees were volunteers who had taken part in the previous Health and Wellbeing Survey conducted by the local NHS board. We sampled to achieve an equal balance of gender and socio-economic backgrounds. Prior to the interviews, participants were given disposable cameras and asked to take photographs of their local areas. Photographs provided a participant determined entry point into a discussion about the quality accessibility and walkability of local facilities and experiences of using urban space. Of the 24 interviewees, fifteen returned photographs prior to interviews. Those who had not were asked either about the photographs they had taken but had not yet developed or what they might take a photograph of. Even when photographs taken did not include local parks or greenspaces, the themes stimulated in discussion proved useful in framing discussion of walkability, access and decisions around the use of greenspace. No incentive was provided in recruiting participants for individual interviews.

The interview schedule was developed through the themes emergent within the participatory focus groups, covered the following;

• Experience of using and accessing local greenspaces.

• Availability of areas for walking, playing and generally being active and how they were used.

• Changes they would like to see.

• How well individuals integrate greenspace use into their daily lives and routines.

• How respondents feel living in their area effects their health and wellbeing.

The researchers did not collect data on how often the interviewees used greenspace but sought to uncover the reasons why different individuals would use, or choose not to use local greenspace.

### Ethical considerations

Participants were recruited from respondents within NHS Glasgow and Clyde's Health and Wellbeing survey who had agreed to be contacted for future studies. As the community sample did not consist of patients, clients or staff of the NHS and involved no invasive procedures, the local NHS Research and Development department waivered the need for ethical approval. Nevertheless, we fore-grounded ethical research practice in the design and conduct of the study including informed consent via opt-in (with ability to leave the study at any time for no specified reason), confidentiality of participant data (pseudonyms are used in reporting), seeking voices not normally heard and stipulating that photography should not include photographs of people.

### Analysis

The visual data (H-diagrams and community maps) were interpreted by the research team and fed into the process of hypothesis formation and the design of interview schedules. In particular, visual data indicated processes and issues over and above those of physical quality and access which presented barriers to greenspace use such as anti-social behaviour, graffiti and conflicts with other greenspace users. The subsequent analysis of interview data was able to explore how individuals experienced and handled such barriers resulting in either the use of non-use of greenspace.

Interviews were recorded and either transcribed or had detailed analytical notes taken by one of the researchers (PS). Analytical notes were combined with materials from participatory groups and the interview schedule to develop an initial coding frame within the *Nvivo *software package [36]. This coding frame was subsequently refined as data was integrated into the data-set. Coding would become hierarchical with time, with variation in a given theme being coded under subheadings of a code. These subheadings could be further divided if required. Analytical codes were checked for salience with the wider research team who had access to transcripts, visual data and had attended participatory groups.

We conducted both within-case analysis, exploring each interview as a case study in greenspace usage and access, and cross-case analysis, looking across all 24 cases for both universal themes and issues and also difference (including deviant cases). Both analyses were conducted iteratively with each informing the other and coded within the same *Nvivo *project.

## Results

Across all interview data, four key categories emerged in shaping decisions around greenspace usage. These were (i) availability of physical community resources (including the provision of greenspace itself), (ii) lifestyle and life-stage factors, (iii) individual values and (iv) levels of felt integratioIn the analysis presented here we draw attention to the emergence of a particular theme: level of perceived integration as a key issue between cases in shaping greenspace use and access.

Although the localities are broadly described as less or more affluent there was socio-economic variation within each area and this diversity is included in our sample. Consequently, a speaker identified as coming from a more affluent area would not necessarily represent 'more affluent' views. Rather than analyse by locality (as indicative of a particular socio-economic status) we analysed individual responses to the experience of using and sharing public space. The influence of socio-economic resources was interpreted as operating at individual level rather than localities.

### Greenspace use, health and wellbeing

Beyond the universal recognition from participants that good quality local greenspace promoted exercise and the taking of fresh air, a range of activities relating to broader wellbeing emerged. Differences in how local greenspaces were perceived to promote health and wellbeing reflected the life-course stage and background of users. Consequently, parents of young children sought safe and pleasant spaces to play, those without dependent children prioritised spaces for socialising with others (the private communal gardens of the North West locality as venues for gatherings an example) and some prioritised the enjoyment of nature. Young people sought places to 'hang out' without being moved on by the police or other adults. The cited relationships between greenspace and wellbeing reflected the different aspirations, expectations and intentions within greenspace use. Consequently, when it came to improvements people wanted to see, alongside universal claims for high quality greenspaces, the promotion of harmonious greenspace use emerged.

Such recommendations could include the installation of CCTV, improved lighting and the return of park wardens or "whistling parkies" as one group referred to them. However, these desires raise a more fundamental question of how community cohesion can be promoted and sustained by public greenspaces in diverse communities were a variety of uses compete in spatially limited spaces.

### Physical availability, quality and access

The availability of good quality greenspace was reported as one of a number of facilitators of use. Indeed many users were able to comment on improvement in greenspace availability and quality over the years. However it must be understood as only one of several factors that lead to use; as necessary but not sufficient.

Well graffiti, it's not too bad (but) there is evidence of it. Litter is another thing although I've noticed the council making more of an effort perhaps even the local residents, who helped clean up last year. That would be my main issue especially down the river walkways. If you had seen it before the trees grew up there it was just plastic bags all down the river. That's probably been the main thing. I think there is a lack of imagination on the greenspaces, I mean they're small areas around here that tend just to be grassed over and that's it.

Rab, more affluent area

They've done quite a lot over the last few years to improve that because I guess it had become a bit sort of smelly and horrible so they dredged the pond and built some grass mounds and things in the middle for swans and so it's a bit nicer looking than it used to be.

Ailsa, more affluent area

### Individual circumstances and values

Alongside quality however, individual life circumstances and associated values needed to configure in such a manner so as to lead to personal preferences and intentions to use greenspace. Quality greenspace would not attract individuals if they had little idea of what to do there. In our data, two value and lifestyle factors dominated the accounts of those who used urban greenspace and these were related to spending time with children and the enjoyment of nature.

Well my kids, my kids and that they all go down the park, they like the park. I played down there when I was younger, when I was a kid I played down there all my life and now it's my weans [children] that's going playing football and all that. It's a good bit, a brilliant bit.

Derek, more deprived area

Not so much now no I don't [use the local park] I probably will because I'm going to have a grand-child soon.

Dolan, more affluent area

The availability of quality greenspace offers a resource that adults and young people could use, which is reinforced by parenting or grand-parenting values that promote active leisure.

We wouldn't let them sit and watch TV, we controlled it a lot, you know. We wouldn't let them have the TV as the focal point all the time you don't want that, you know. A lot of kids, maybe that's all for them nowadays too, maybe they're just couch potatoes, you know. It makes them lazy too, and it's an unhealthy lifestyle.

Dolan, more deprived area

### Social cohesion and problems in encountering other greenspace users: self-removal from public space

The presence of young people in parks (although identified as appropriate users) could produce ambivalent feelings from respondents. Many found the presence of unsupervised, older children and adolescents a barrier to greenspace. This finding highlights how community cohesion becomes an important factor in determining greenspace access. The perceptions subgroups (such as adults and young people) have of one another can lead to self-exclusion of some (such as adults) from parks. This self-exclusion becomes an important barrier to greenspace access not captured in understandings of physical quality of greenspace. For Archie (below) accessibility was impeded by the presence of younger people.

They could make it a bit more accessible, I don't mean getting there, but I mean it's inhabited by the youngsters now and they're not very friendly youngsters, you know, it can be quite intimidating at times, you know. I think perhaps if they put more effort into maybe policing that park then you'd probably get people having a walk round it. I would rather travel anyway, you know, for a walk I would rather travel somewhere else, you know.

Archie, more deprived area

The presence of young people could be associated (fairly or unfairly) with anti-social behaviour. There were two responses to young people and the (related) issues of graffiti and perceived incivility by adults in the study. These responses appear to have origins in the level of integration and confidence individuals felt in their communities.

One response was fear of young people in public space, leading to either a removal of oneself and family from public space or, for those with children, to an increase in the amount of supervision felt necessary. For those who had more resources and choices, exiting public space for private resources was a more easily achieved strategy. The second response was to be stoical about the presence of anti-social behaviour. Stoicism appeared to be grounded in being confident about oneself and having a degree of control.

Jack, a fifty-seven year old carer in the North, chose to remove himself from public space on the basis of his experiences with others in his local community. Despite living in the area for thirty years, he felt unable to use the local public greenspaces on account of fears about those he described as *"drug users, neds and yobs". *When Jack wanted to walk in greenspace, he would take a train to the coast 20 or so miles away. After Jack's home was burgled, he installed closed circuit television cameras reflecting a lack of confidence in his wider community and societal responses to crime. He felt stiffer law and order responses and more police on the beat were required. In terms of local leisure opportunities, Jack chose to join a local private bowling club. While he did not possess a high disposable income, he felt the private club was the only means of securing his safety locally.

It's out of control. Law and order has broken down. All they do is put a veneer on it. They use all these initiatives, it's all whitewash, the criminal justice system has broken down ... It's private members' club so you don't get..., it run by ex army and there is a duty officer on and he's responsible for discipline, anybody steps out of line, raising their voice, swearing, anything like that, you're on a charge and they're stricter than the courts. I mean some of them have been sin binned, six months suspension.

Jack, more affluent area

Jack's choice to use private greenspace facilities reveals that for him, accessibility is not an issue of quality but wider societal issues of cohesion and of shared values. What the private space provides is certainty, viewed as absent in public space, about the values and behaviours with which he will come into contact.

### Social cohesion and problems in encountering other greenspace users: increased supervision of children

Naomi dealt with the issue of anti-social behaviour in shared public space differently, choosing to increase the monitoring and supervision she gave to her children to enable them to play outside.

Naomi: You're feert (scared) to let your kids out on the streets you know the way we used to go out and play, that doesnae happen here.

Interviewer: Do you mean because of the traffic or just because of the vandalism or...

Naomi: Just because of everything, because of people hanging about and the threat of somebody maybe taking them away or all the fighting and things like that, that go on.

Interviewer: It's obviously quite frustrating for you.

Naomi: Well, as I say I just take them out myself, if they go out on their bikes we go out with them as well.

Naomi, more deprived area

Exploration of Naomi's circumstances reveals how she also chose to remove herself and her children from shared public space on account of safety fears. As with Jack, it was not the quality of resources available that prevented her usage, it was the overriding sense that the values and behaviours she found in her community around her were not ones that would ensure safety. Neither was Naomi particularly socio-economically disadvantaged; indeed her access to resources such as a car enabled the removal of herself and children from community resources such as parks, ferrying her children to organised activities such as dancing lessons. The theme of controlling the circumstances, people and values her children came into contact with pervaded.

Another individual who selected her public space use with care was Nyela, a female asylum seeker from Somalia, in her twenties with three young children. Nyela differed from Jack and Naomi in a number of ways, not least amongst them that she was socio-economically more disadvantaged and she described the greenspace she could access as poor quality. The data collected from her came from a discussion group using participatory methods. Nyela and a group of fellow migrant women drew maps that revealed their perceptions of greenspace available to them. Objectively, the area they lived in was well provided for in terms of facilities, a country park was adjacent to the area they lived in. However the greenspace maps drawn by Nyela and her friends depicted only football pitches and low quality green space (open plains of grass) that flanked the high rises in her neighbourhood. The maps also listed a number of obstacles to greenspace usage such as broken lifts, racist graffiti, gangs of young people "shouting things" and the tendency for flooding after rain (frequent in the West of Scotland).

The experience of Nyela and her fellow asylum seekers offers an important counterpoint to the earlier findings about the role of children in promoting greenspace access. Having young children did not always result in use of free amenities such as local parks, as fear of navigating local communities remained a significant barrier. Chosen instead was the local community café, established by a community health project, because it provided feelings of safety and inclusion.

### Stoical responses - strategies to normalise neighbourhood incivilities

The second type of response to evidence of anti-social behaviour in public space we coded as stoical responses. These were responses where people recognised the presence of incivilities and anti-social behaviour and noted the deleterious effect it had on the experience of using urban greenspace and public space in general. However, often through attempts to understand the origins of such behaviours, stoics were able to perceive the actions less threateningly. Moira, below, when reporting anti-social behaviour she has suffered attempted to play down its severity (in italics).

A couple of things, we've had a bit of a spate of vandalism *and I know it's fairly low level vandalism *it's not as if they are drawing all over our walls or something like that, but there's been some tagging with, I believe that's what they call it, tagging ... *So that's a bit disappointing *and also about a month ago somebody broke into my dad's car when it was parked outside the house and it happened in the middle of the day. *That was disappointing that that happened. It's low level it's not anything*, *it's not anything that I'm going to get particularly upset about *I think *it's an annoyance it's not something I get really, really bogged down *[with] and, I was very annoyed obviously about my dad's car getting broken into, but in terms of things like the litter and the vandalism *I think it's something that happens when you live in a city*.

Moira, more affluent area

Attempts to understand the causes of such incivilities and anti-social behaviour can be interpreted as attempts to maintain a sense of shared values and social cohesion in light of evidence of its possible decline. Such responses are in many ways more hopeful than the previous fearful responses, as the response itself does not further exacerbate the separation of young and old in public space.

Yeah, maybe, *but I'd like to think it's more that I can understand why these things happen*, things like vandalism happens why do we drop litter and hang about streets and stuff like that because *they've probably just not got anything else to do and they're bored and fed up *and that's just how you find a lot of areas like Glasgow and I think you just accept that *that could have been me if maybe I hadn't had the same advantages and I don't like to judge people*. Yes I would be annoyed if somebody sprayed painted my windows or something like that, but I'd like to think that once I'd calmed down I could say well *there might be a reason why that happened and it's maybe not through fault or it's just a collection of circumstances have led to that *and we've all got a part to play really in trying to make sure these things don't happen and people don't really need to do that.

Moira, more affluent area

A similar logic is in operation in the thinking of Kevin, a middle class parent in the more affluent part of the city. He comments on the presence of young people in public space in more positive terms, even taking account of associated incivilities such as graffiti and behaviour that can often be interpreted as rowdy or potentially threatening. He refers to a local skate park where young people engage in leisure pursuits as offering colour and vibrancy to public space (even with the associated graffiti). He finds the growth of corporate monopolies in his community and the homogenisation of his high street to be more anti-social. Difference for Kevin is to be celebrated and encouraged and a benefit of living in a mixed urban community. Kevin is a keen cyclist and therefore a frequent user of public space and perhaps more able to engage (albeit fleetingly) with its many aspects.

## Conclusion and Discussion

A recent study exploring relationships between environment and obesity in a Canadian city cited a cultural preference for car ownership and use as reducing the potentially beneficial effects of the city's good greenspace quality [37] Such a finding illustrates how elements of local cultures that may promote use of urban greenspace feature combinations of objective and subjective factors. Objectively demonstrable conditions (such as provision of infrastructure and greenspace) are experienced through subjective and inter-subjective 'rationalities' around the appropriateness of using greenspace as a leisure choice or in daily life.

Our analysis adds to this developing understanding by highlighting the role of social cohesion subjectively experienced as how we anticipate and interpret the actions of others in public space. Consequently social cohesion should be understood as an important component of accessibility around urban greenspace.

The quality of the greenspace on offer interacts at subjective with the level of social cohesion perceived within a community. Whether the greenspace 'offer' made through infrastructure and provision coincides with individual values and their resulting motivations is an individual level factor that also shaping access.; However, access to personal resources also underpin a sense of social inclusion, when personal resources mitigate the physical evidence of low levels of social cohesion in a community (such as graffiti and other evidence of anti-social behaviour).

Where a feeling of social inclusion was absent, the self-removal of individuals from community greenspace resources could be observed. In the cases of those with fewer resources, the removal of an accessible and free at the point of use community resource such as public parks can compound the material disadvantages that underlie population level health inequalities. The findings therefore illustrate the role of social capital, particularly the components of trust, networks of cooperation and strong community identity, in facilitating, access to a health resource and its central role in maintaining wellbeing for all members of the geographical community [38].

The findings indicate support for previous work suggesting aspects of local environments that heighten feelings of insecurity may be mechanisms through which place affects health [39]. One process through which this is played-out for individuals in a manner that impedes access to urban greenspace, is through anxiety about others. Previous research investigating resident's perceptions of walkability in neighbourhoods has found that safety and crime attributes were not as significant as objective, physical attributes in creating a sense of walkability [40]. However, our study, being qualitative, allowed for investigation of how evidence of crime and anti-social behaviour is interpreted by potential greenspace users. This process can be understood as a meeting of a cultural component; the decline in trust and social cohesion between groups (in this case, intergenerational) and the individual circumstances which shape subjective orientations toward the cultural circumstances at large; feeling confidence that stems from greater social inclusion. Previous research has identified the presence of others as a key facilitator of greenspace use, particular for women however, a decline in trust in communities can make the presence of others a barrier to use and is not always interpreted positively [41].

This local cultural aspect of neighbourhoods or urban areas should be considered alongside quality, availability and connectedness of space ('walkability') and the characteristics of populations. Levels of social cohesion appear a key process variable by which inequalities in access can continue for certain marginalised subgroups once established. In the context of this study, this is experienced as an intergenerational segregation of public space use, between younger and older users. In other contexts the absence of community cohesion may be evident in other ways between different groups, possibly evident through ethnic, gang associated or sectarian division.

Segregation in the use of public space highlights one way in which low levels of community bridging social capital mediates individual health inequalities previously identified by researchers such as Cattell and how those with more resources can protect themselves from some of the health consequences stemming from reduced community cohesion [42]. Addressing such an issue currently appears outside the scope of urban planning given that broader societal aspects that underpin feelings of social cohesion are only partly remedied by the provision of community enhancing physical environments. However, given that the inclusion of diverse groups in public spaces can potentially enhance social cohesion, opportunities are currently being missed. As the expertise and resources required to improve the diversity of patronage of our urban greenspaces falls outside the traditional skill sets of urban planners, we suggest the continued and improved involvement of community members and their grassroots organisations, as well as other statutory bodies, in the development of urban greenspace planning and implementation. In some contexts, this may involve conflict-resolution efforts as much as the provision of high quality infrastructure and greenspace.

## Competing interests

Russell Jones is a Board Member of the Glasgow and Clyde Valley Green Network

## Authors' contributions

PS designed the qualitative component of the FAB greenspaces study, oversaw and participated in data collection, led on the analysis and interpretation of data and is responsible for the drafting, and argument found within, this manuscript. RJ managed the broader *FAB greenspaces *study and made contributions to study design, data collection, analysis and drafting of the manuscript. AE was involved in the steering group around the qualitative study, made contributions to analysis and the drafting of the manuscript. All authors read and approved the final manuscript.
